# A meta-analysis of functional reading systems in typically developing and struggling readers across different alphabetic languages

**DOI:** 10.3389/fpsyg.2015.00191

**Published:** 2015-03-10

**Authors:** Courtney Pollack, Gigi Luk, Joanna A. Christodoulou

**Affiliations:** ^1^Harvard Graduate School of EducationCambridge, MA, USA; ^2^MGH Institute of Health ProfessionsBoston, MA, USA

**Keywords:** ALE meta-analysis, reading development, alphabetic languages, typical readers, struggling readers

## Abstract

Functional neuroimaging research has identified multiple brain regions supporting reading-related activity in typical and atypical readers across different alphabetic languages. Previous meta-analyses performed on these functional magnetic resonance imaging findings typically report significant between-group contrasts comparing typical readers and readers with reading difficulty or a clinical diagnosis of developmental dyslexia. In order to advance our understanding of cross-linguistic convergence of reading-related brain activations for these reader groups, analyses using activation likelihood estimation were carried out separately for typical and atypical readers who ranged from children to adults. Contrasts were analyzed for tasks involving rhyming or reading of letter or word stimuli presented visually in English, Dutch, Italian, German, French, or Norwegian. Typical readers showed reliable activation in only left lateralized regions, including the inferior frontal area, precentral area and middle temporal gyrus. Atypical readers also showed activation in the left inferior frontal area and precentral region, in addition to significant activations in the right hemisphere, including the superior, medial and inferior frontal regions, lingual gyrus and the inferior occipital area. These results distinguish between typical and atypical reader group activations, showing common and distinct regions of activation when engaged in reading-related activities, extending previous meta-analyses on identifying brain regions relevant to reading to include cross-linguistic analyses for alphabetic scripts. Results support the universality of a signature pattern of brain activation in developmental dyslexia across alphabetic languages.

## INTRODUCTION

Reading involves translating written symbols to sounds in order to extract meaning. This process is complex, and to be fluent, readers coordinate multiple skills such as word decoding and reading rate effectively and simultaneously so that they can attend to comprehension demands from the text (LaBerge and Samuels, 1974; Stanovich, 1986). Given the complex sensory and cognitive mechanisms involved in reading, it is not surprising that substantial behavioral variability is observed across reading development. In particular, a significant portion of school-age children experience difficulty in reading acquisition. In the United States, as many as 15–20% of the school-age population show evidence of difficulty in reading (Moats and Dakin, 2008). Significant research effort has been dedicated to understanding individual differences in reading development, including multiple perspectives of research spanning social (e.g., Schaffner et al., 2013), cognitive (e.g., Fuchs et al., 2012), genetic (e.g., Astrom et al., 2012), and brain activation patterns (e.g., Yamada et al., 2011). For functional neuroimaging research in particular, accumulating research has established a set of brain regions that has been shown to support reading-related activities in typical readers in contrast to struggling readers (for a review, see Gabrieli et al., 2010; Blomert, 2011).

The present study aims to extend these findings by examining cross-linguistic convergence of brain activation patterns for typical readers and struggling readers separately, rather than comparatively. To date, empirical evidence largely contributes to our understanding of brain regions that are engaged in typical readers versus struggling readers, which offers a comparative perspective on brain activations for reading. The focus of these analyses is to identify the common and distinct brain regions that contribute to reading in typical readers and in struggling readers. To expand our understanding of reading brain systems, the current study examines brain systems engaged for typical readers and struggling readers as distinct groups. With the aim to examine cross-linguistic convergence, studies conducted in different alphabetic languages and countries are included in this meta-analysis. These studies have variable inclusionary criteria for struggling readers. In the present paper, the term *struggling readers* refers to children and adults fitting one or more of the following criteria: (1) reported familial risk of reading difficulty; (2) received a clinical diagnosis of dyslexia; (3) showed significantly lower performance in reading-related tasks (e.g., 1 or 2 SD below average on standardized measures) and the low performance cannot be attributed to impoverished learning opportunity, dysfunctional visual or auditory processing and inferior intelligence.

Converging evidence across functional magnetic resonance imaging (fMRI) studies indicate a distinct brain activation pattern for reading. Early in reading development, children show activations in bilateral regions in temporo-parietal, temporo-occipital, and inferior frontal regions (Yamada et al., 2011). During early elementary school years, typically developing readers shift from bilateral to left lateralized recruitment of these regions (Gabrieli et al., 2010). This pattern is relatively stable into adulthood, with the anterior system supporting motor production and the processing of low-frequency exception words and non-words; the posterior dorsal system supporting grapheme–phoneme correspondence and efficient word reading; and the posterior ventral system supporting automatic recognition of printed words (Fiez and Petersen, 1998; Cohen et al., 2000; Shaywitz et al., 2002). Struggling readers, most often characterized with developmental dyslexia, show a distinct brain activation pattern that relies on right hemisphere homologous regions in the posterior temporo-parietal and temporo-occipital regions (Rumsey et al., 1992, 1997; Shaywitz et al., 1998, 2002; Brunswick et al., 1999; Simos et al., 2000; Paulesu et al., 2001). Struggling reader groups have also shown hyperactivation of frontal regions (Shaywitz et al., 2002), although evidence suggests this increased activation is not a signature of dyslexia but rather a reflection of the increased difficulty of the task given similar activations in dyslexic children and reading-matched peers compared to age-matched peers (Hoeft et al., 2007). The signature brain activation pattern has been shown to be independent of cognitive abilities (i.e., IQ; Tanaka et al., 2011) and consistent for native readers of different alphabetic script-based languages (Paulesu et al., 2001). Furthermore, struggling readers who show reading improvement following reading intervention show activation patterns that more closely approximate that of their typically developing peers (e.g., children: Temple et al., 2003; adults: Eden et al., 2004).

In an effort to summarize neuroimaging findings comparing sets of brain regions supporting the processing of reading-related tasks, meta-analysis has been carried out on between-group contrasts comparing typical and struggling readers (Maisog et al., 2008; Richlan et al., 2009, 2011). These meta-analyses provide an overview of brain activation patterns across samples in different studies that share similar experimental procedures, including participant inclusionary/exclusionary criteria and tasks. Using between-group contrasts, Maisog et al. (2008) characterized regions of hypo- and hyperactivation that were consistent across studies involving struggling readers with dyslexia across different alphabetic languages. Maisog et al. (2008) reported that typical adult readers exhibited higher activation than struggling adult readers in a large set of brain regions, including the bilateral inferior frontal gyrus, left inferior parietal gyrus, right postcentral gyrus, bilateral fusiform gyrus, bilateral superior temporal gyrus, thalamus, left precuneus and left middle occipital area. In contrast, struggling readers showed higher activity in the right insula and right thalamus. In the case of adult readers, the difference in activation patterns converged in the hypoactivation in the left hemisphere. This finding was consistent with subsequent research in children reporting a lack of engagement in the left temporal and occipital regions (e.g., Shaywitz et al., 1998, 2002; Temple et al., 2001) and possible neural response to behavioral intervention that resulted in improved reading skills (Temple et al., 2003) or a compensatory mechanism to overcome challenges in reading (Shaywitz et al., 2003).

Richlan et al. (2009) sought to provide more specific locations for activation abnormalities that had previously been characterized broadly as temporo-parietal and occipito-temporal. Consistent with the Maisog et al. (2008) findings, Richlan et al. (2009) reported that struggling readers, combining both children and adults, demonstrated hypoactivation in inferior frontal, parietal, and temporal regions in the left hemisphere and hyperactivation in left subcortical regions and right medial frontal area. Subsequently, Richlan et al. (2011) conducted two meta-analyses separately for children (ages ranged from 9 to 11) and adults (ages ranged from 18 to 30). In these analyses, contrasts between typical and atypical readers were analyzed with Signed Differential Mapping (SDM) software, which combines features of activation likelihood estimation (ALE) and another meta-analytic method, multilevel kernel density analysis (MKDA; for a discussion, see Radua and Mataix-Cols, 2009). Across these studies, struggling children readers showed hypoactivation in the bilateral inferior parietal lobules and no hyperactivation in any brain region. In contrast, struggling adult readers showed significant hypoactivation in the left fusiform gyrus and hyperactivation in bilateral subcortical areas. These cross-sectional findings comparing typical and struggling readers further suggest a lack of engagement in left temporal and occipital regions in supporting fluent and accurate reading-related activities in adults and these regions were not readily recruited to support reading in children.

The above meta-analyses focus on between-group contrasts and thus are helpful in understanding *divergent* brain activation patterns between-groups. However, these prior meta-analyses do not speak to the *convergence* of brain activation between-groups. To address this gap in the literature, the present meta-analysis examines brain regions recruited to engage in reading-related tasks separately for typical and struggling readers. Unlike previous meta-analyses, we used meta-analysis with the goal of identifying both common and distinct brain regions in children and adults with varying reading ability across different alphabetic script-based languages. This approach can help identify clusters that can be used in future connectivity analyses to illuminate the interaction between brain regions as an interconnected network that supports reading. Specifically, selecting brain regions that are common to both typical and atypical readers could inform understanding of the common and differential functional networks that are employed in readers with diverse reading capacity. To this end, we included published studies that reported activation coordinates separately for typical and struggling readers. Two meta-analyses were conducted separately for these two groups of readers. Given that children are in the process of developing fluent reading and that engaging in reading-related tasks is effortful, it is expected that typical and struggling readers would recruit left frontal regions when engaging in reading-related tasks. However, we expected typical and struggling readers to demonstrate different activation patterns in posterior brain regions because of struggling readers’ atypical mapping of sound-symbol correspondence.

## MATERIALS AND METHODS

### STUDY SELECTION AND PARTICIPANTS

An initial PubMed database search with search criteria: “dyslexia” or “reading difficulty” and “fMRI” or “PET” yielded 508 potential papers. These papers were then reviewed against a set of inclusion criteria. Papers included in the analysis were published in English, used fMRI or PET methods, used whole brain analyses, reported foci separately for typical and atypical readers who had matched demographics, included visual tasks that involved letter or word stimuli, and involved alphabetic languages. Case studies or studies involving clinical populations (e.g., patients with schizophrenia) were excluded. Studies reporting region-of-interest (ROI) analyses were also excluded. A final set of 13 papers contributed 131 foci for typical readers and 101 foci for atypical readers (see **Table 1**).

**Table 1:** List of foci, number of experiments and subjects, by reader type.

Reader type	Foci	Number of experiments	*n*
Typical	131	13	172
Struggling	101	13	174


Using this set of inclusion–exclusion criteria, we were able to obtain two samples of typical and struggling readers with comparable sample sizes and matched demographic characteristics. In total, there were 172 typical readers and 174 struggling readers across the 13 studies (see **Table 1**). In one study, Hoeft et al. (2006) examined both age-matched and reading-matched typical readers as control participants relative to atypical readers. Age-matched typical readers were included rather than reading-matched typical readers to maintain consistency across the set of studies. Across these studies, struggling readers were characterized as having reading difficulties or dyslexia based on either previous diagnoses (including familial risk assessment) or behavioral evaluations for inclusion in that group for the specific study.

**Table 2** provides an overview of each study, including participant demographics, task descriptions and criteria for participants to be classified as struggling readers. Studies spanned six different alphabetic languages: English, Dutch, Italian, German, French, and Norwegian, which are comparable to studies included in previous meta-analyses. Studies included child, adolescent, and adult participants (i.e., about 8–63 years). Ten studies examined child and/or adolescent participants, two studies examined adult participants, and one study examined participants from adolescence into adulthood.

**Table 2:** Characteristics of studies included in the meta-analysis.

Study	Reference	Language	*N*	Experimental task	Baseline task	Statistical threshold^a^	Age range (years)	Struggling reader criteria^c^
1	Paulesu et al. (1996)	English	11	Rhyme letters	Match symbols	*p* < 0.001	25.20 – 27.20	H, C
2	Temple et al. (2001)	English	39	Rhyme letters	Match letters	*p* < 0.025	8.60 – 11.60	H, L
3	Cao et al. (2006)	English	28	Rhyme words	Fixation	*p* < 0.001	8.80 – 14.11	H, L
4	Cao et al. (2008)	English	24	Rhyme words	Fixation	*p* < 0.001	12.30 – 14.11	H, L
5	Hoeft et al. (2006)	English	30	Rhyme words	Fixation	*p* < 0.001	8.00 – 12.00	L
6	Backes et al. (2002)	Dutch	16	Rhyme pseudowords	Fixation	*p* < 0.05	11.00 – 12.00	H, C, L
7	MacSweeney et al. (2009)	English	14	Rhyme pictures	Match pictures	*p* < 0.05 voxelwise *p* < 0.005 clusterwise	18.05 – 48.06	H
8	Brambati et al. (2006)	Italian	24	Read words	View false font strings	*p* < 0.005	13.00 – 63.00	L
9	Georgiewa et al. (1999)	German	34	Read pseudowords	View false font strings	*p* < 0.05	9.00 – 17.00	L
10	van der Mark et al. (2009)	German	42	Lexical decision	View false font strings	*p* < 0.001	9.70 – 12.50	L
11	Monzalvo et al. (2012)	French	46	View word pairs	Blank	*p* < 0.05, corr.	8.90 – 10.80	H, C, L
12	Peyrin et al. (2011)	French	24	Categorical matching	Fixation	*p* < 0.001	10^b^	H, C, L
13	Beneventi et al. (2009)	Norwegian	24	Match letters	Blank	FDR < 0.05	13^b^	H, L

*^a^Statistical thresholds are uncorrected, unless otherwise noted.*

*^b^Mean age reported only.*

*^c^H, documented history or diagnosis of dyslexia; C, Clinically referred reader with dyslexia; L, Low score (e.g., one or two SD below the norm, age-adjusted) on standardized test(s) of reading (e.g., Woodcock Reading Mastery Test measures).*

### TASK DESCRIPTION

Eight of the 13 studies (see **Table 2**, studies 1, 2, 6, 8–10, 12, 13) separately reported more than one contrast for both typical and struggling readers, such as an activation condition (e.g., rhyming words or letter names) versus baseline (e.g., fixation, blank) and an activation condition versus a control condition (e.g., matching letters, matching symbol strings). Almost all studies required a button press (see **Table 2**, studies 2–8, 10–13); one study required movement of a joystick (1) and one did not require a motor response (9). In accordance with prior meta-analyses involving participants with dyslexia (e.g., Richlan et al., 2009, 2011), only one contrast per reading group was included from each study. As in Richlan et al. (2009), preference was given to contrasts that involved phonological tasks such as rhyme judgment, reading words, or reading pseudowords. When more than one contrast involved phonological tasks, the task with the higher number of reported foci was used. Seven studies included an experimental task involving rhyming of letters, words, pseudowords, or pictures. During rhyming tasks, participants determine whether two visually presented alphabetic stimuli (e.g., letters, words) rhyme. As control tasks, participants either experienced a rest condition (e.g., fixation) or completed a matching task (e.g., determine if two letters match). In two studies (Georgiewa et al., 1999; Brambati et al., 2006), participants were asked to read silently either words or pseudowords (i.e., pronounceable letter strings with no meaning). Baseline tasks involved viewing false font strings (e.g., strings of non-alphabetic characters). Study 10 (van der Mark et al., 2009) used a phonological lexical decision task, in which participants determined whether a visually presented stimulus sounded like a word. Study 11 used passive viewing of word pairs, in which participants attended to pairs of four-letter common French words (Monzalvo et al., 2012). Participants responded to stars interspersed throughout blocks to maintain visual attention on the word pairs. Study 12 used a categorical matching task with letters and geometric figures, in which participants responded when two visually presented images were unmatched (e.g., one letter and one geometric figure; Peyrin et al., 2011). Finally, in study 13 participants completed a letter match task in which they first read silently a string of six lowercase letters and then determined if a visually presented pair of letters, one upper case and one lower case, matched (Beneventi et al., 2009).

### DATA ANALYSIS

Two meta-analyses were conducted using ALE: one for typical readers and one for struggling readers with matching demographics. Both meta-analyses were conducted using GingerALE version 2.1.3 (Eickhoff et al., 2009, 2012; Turkeltaub et al., 2012). Coordinates for each study were reported in either MNI or Talairach space. Prior to analysis, MNI coordinates were converted to Talairach space using the *icbm2tal* transform provided in the GingerALE software (Lancaster et al., 2007).

GingerALE was first developed by Turkeltaub et al. (2002) and updated with a revised algorithm by Eickhoff et al. (2009). ALE treats foci as three-dimensional Gaussian distributions that are centered on the reported coordinates (Eickhoff et al., 2009; Turkeltaub and Branch Coslett, 2010) and computes the union of activation probabilities for each voxel to get ALE maps. At each voxel, the ALE maps are compared to an ALE null distribution, which has been determined by a permutation test, to obtain associated *p*-values. A resulting threshold for the ALE map is computed based on the chosen false discovery rate (FDR; Laird et al., 2005). A minimum cluster size of 100 mm^3^ was used to create a thresholded ALE map for each meta-analysis and for subsequent cluster analysis with FDR = 0.01. All results were obtained using the non-additive method, which limits within-experiment effects (Turkeltaub et al., 2012). Results were reported in Talairach space (Talairach and Tournoux, 1988), displayed using the anatomical templates provided by the GingerALE program, and labeled using the Talairach Daemon in Mango (Lancaster and Martinez, n.d.).

## RESULTS

The results of the two meta-analyses are reported in **Table 3**. Converging activated brain regions for typical readers are reported on the top panel of the table. Consistent with previous research, typical readers showed activation in left frontal and temporal regions when engaging in reading-related tasks, with the largest clusters observed in the left inferior frontal gyrus (LIFG; BA 44) and left precentral gyrus (BA 6). Since half of the studies included in the analysis were conducted in English, we further investigated whether the reported regions were biased toward English, which has a higher entropy between phonemes and graphemes relative to other alphabetic languages in the analysis (Borgwaldt et al., 2005; Ziegler et al., 2010). The ALE output includes a list of contributing studies, which indicate studies reporting foci within the boundary of the cluster. In the last column of **Table 3**, studies conducted in non-English languages were italicized. For each cluster reported in the top panel of **Table 3** for typical readers, studies conducted with English and non-English languages were represented, indicating cross-linguistic convergence that supports reading-related processes in alphabetic languages. However, in the lower panel, studies conducted in English and non-English languages appeared to represent different regions.

**Table 3:** Activation likelihood estimation results, including cluster, Talairach coordinate, ALE value, volume, and contributing studies for typical and struggling readers.

	Talairach coordinates					
Cluster	*x*	*y*	*z*	ALE value	Volume (mm^3^)	Contributing studies
**Typical readers**
1. Left inferior frontal gyrus (BA 44)	-46	12	10	0.02075	1784	1, 3, *8*^a^*, 9, 10*
2. Left precentral gyrus (BA 6)	-42	2	32	0.01848	1032	1, 3, 4, 7, *11*
3. Left fusiform gyrus (BA 37)	-38	-44	-14	0.01664	560	*8, 12,* 4
4. Left superior frontal gyrus (BA 6)	-2	12	52	0.01225	528	3, *8, 13*
5. Left–middle temporal gyrus (BA 22)	-58	-38	4	0.01404	296	4, *8*
6. Midline cingulate gyrus (BA 32)	0	22	38	0.01277	176	5, *12*
**Struggling readers**
a. Left insula (BA 13)	-34	22	10	0.01649	552	1, 2, 4
b. Right lingual gyrus (BA 18)	34	-72	-8	0.01551	496	2, 3, 7
c. Left inferior frontal gyrus (BA 44)	-48	12	12	0.01413	440	2, 5, *8*
d. Right inferior occipital gyrus (BA 18)	28	-86	-6	0.01340	352	4, *6, 13*
e. Left precentral gyrus (BA 6)	-56	-2	32	0.01261	304	3, 4, 5
f. Right superior frontal gyrus (BA 6)	2	8	48	0.01151	240	7, *8*
g. Left medial frontal gyrus (BA 6)	-10	-2	54	0.01447	240	2, 4
h. Left–middle occipital gyrus (BA 19)	-38	-72	-8	0.01281	160	3, 4
i. Left–middle frontal gyrus (BA 46)	-46	20	20	0.01145	160	*8, 10*
j. Right medial frontal gyrus (BA 6)	6	16	42	0.01123	120	None reported
k. Left inferior frontal gyrus (BA 9)	-36	8	22	0.01080	112	*13*
l. Right inferior frontal gyrus (BA 9)	48	16	20	0.01101	104	*8, 13*
m. Left superior parietal lobe (BA 7)	-28	-64	48	0.01140	104	*6, 13*


*^a^Italicized numbers indicated studies that were conducted in non-English languages. These numbers correspond to study number in **Table 2**.*

As shown in the lower panel of **Table 3**, struggling readers across studies showed a more distributed set of regions showing significant activations when engaging in reading-related tasks. In total, 13 clusters showed reliable activation for atypical readers. Importantly, activation is reliable even though some clusters show only a small number of contributing studies (e.g., cluster j, cluster k). Because contributing studies are only those that report foci within the boundary of the cluster, additional studies that are not listed may have contributed foci that are located near and just outside of the cluster boundary (Fox et al., 2013). In addition, the statistically significant clusters identified here meet a stringent statistical threshold with FDR corrections (FDR = 0.01) to guard against false positives.

Unlike the left lateralized regions in frontal and temporal lobes observed in the typical readers, struggling readers demonstrated activation in both left and right brain areas, covering frontal, temporal, parietal, and occipital regions. Collectively, the largest cluster was observed in the left insula (BA 13) and the second largest cluster was in the right insula (BA 18). These two largest clusters were much smaller in volume compared to those reported for typical readers. No study was reported as contributing to the right medial frontal gyrus (BA 6; cluster j). The lack of contributing studies indicated no reported focus was within the boundary of this cluster, although as described above, there may be foci reported surrounding the significant cluster. Therefore, the lack of contributing studies does not invalidate the results. In regards to the potential bias of contributing studies based on language of administration, studies conducted in English seem to contribute to larger clusters.

**FIGURE 1:**
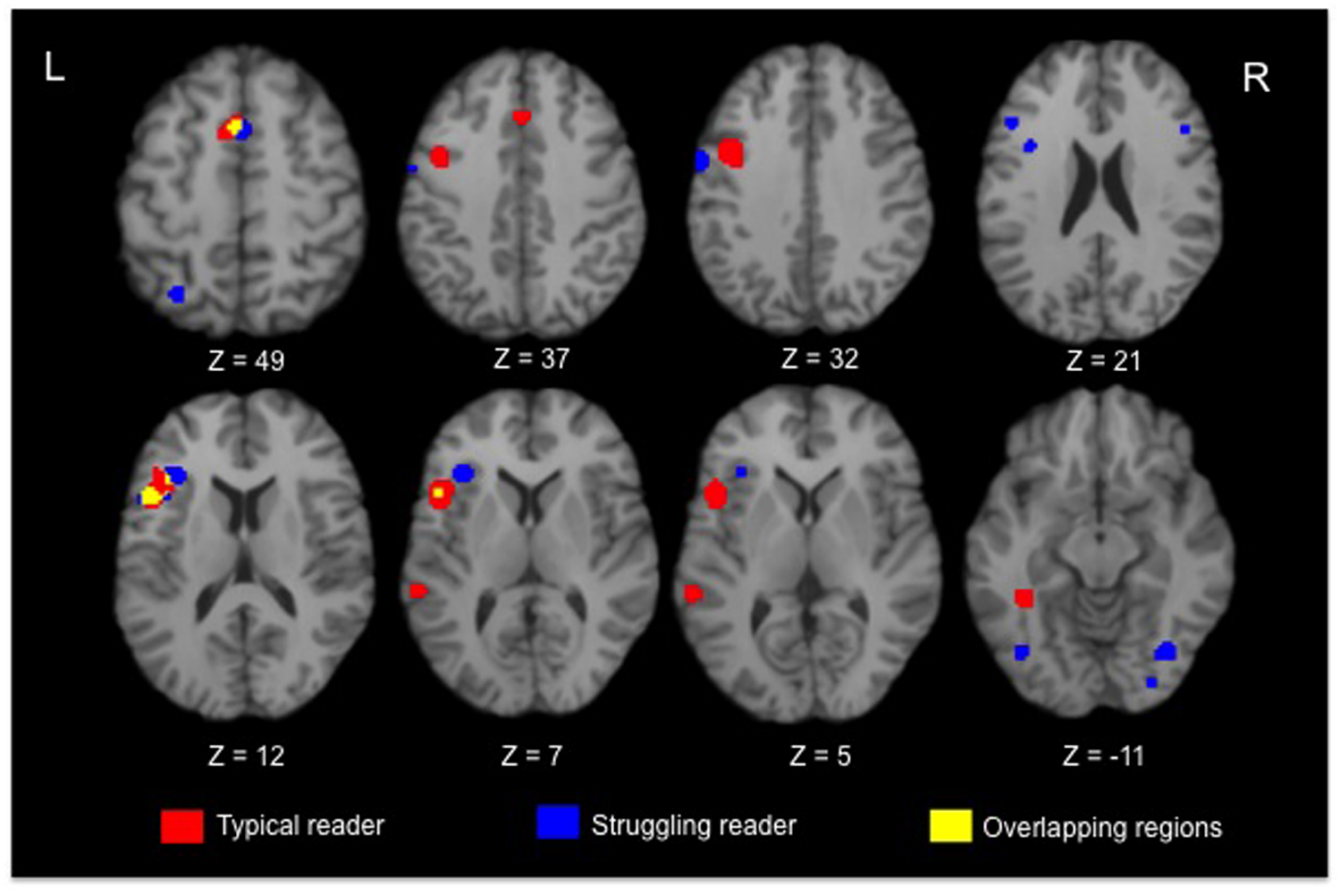
**Selective axial slices showing clusters of brain regions with significant activation in typical (red) and struggling readers (blue).** The overlapping clusters showing converging regions of activation are in yellow.

Significant activated regions for typical and struggling readers are shown in red and blue, respectively, in **Figure 1**, with the overlapping regions coded as yellow. Two converging regions were observed between typical and atypical readers: the LIFG (BA 44) and the left superior frontal gyrus (BA 6). Aside from these frontal regions, no converging regions were observed in the posterior brain regions. In terms of regions showing divergent activation patterns, struggling readers recruited bilateral frontal regions, parietal and occipital regions when completing reading-related tasks while typical readers showed robust activation in the inferior frontal and fusiform gyrus (BA 37).

## DISCUSSION

The present study examined the distinct and overlapping brain regions in typical and struggling readers when engaging in reading-related tasks. Using ALE, 13 studies involving matching typical and struggling readers were included in the present meta-analyses. Previous work has focused on between-group contrasts of activation, highlighting particular brain regions with hyper- or hypoactivation in struggling readers compared to typical readers. The present report included separate analyses for typical developing and struggling readers. Unlike between-group contrasts of activation, these separate analyses surfaced individual activation patterns for typical and struggling readers across brain regions, respectively. In reporting these patterns separately for each group, we were additionally able to qualitatively compare brain regions across groups of typical and struggling readers. Importantly, in addition to identifying distinct regions of activation, we were able to examine common regions of shared activation between typical and atypical readers. Three major findings were observed: (1) Typical readers showed left lateralized activation in frontal and temporal areas when engaging in reading-related tasks; (2) Struggling readers showed distributed bilateral activation patterns in frontal, temporal, and occipital regions; and (3) Both typical and struggling readers showed activation in the left inferior frontal area and in the precentral area. We consider the implications of each of these results in turn.

As a complex behavior that takes years to develop and acquire, reading relies on a set of brain regions to orchestrate coherently. Consistent with previous research and meta-analysis, left lateralized regions were observed to have reliable activation across studies in the typical readers, including inferior and superior frontal regions and associative temporal areas (e.g., Jobard et al., 2003; Turkeltaub et al., 2003; Houdé et al., 2010; Sebastian et al., 2014). This left lateralized network reflects a set of brain regions that gradually specialized in supporting reading-related behavior. In addition to the left lateralized regions, typical readers showed reliable activation in the precentral gyrus, potentially reflecting the demand to control and produce motor responses during tasks. Given that the sample of studies in this analysis involved typical readers who are at least in middle childhood (at least age 8) and that the majority of the tasks described in **Table 1** involved phonological processing, it is reasonable to believe that the results observed in the typical readers were indicative of a relatively secure phonological processing system, which is an important component to reading success in alphabetic languages.

The second analysis involving struggling readers showed a bilaterally distributed activation pattern, which is consistent with previous research using empirical functional connectivity analysis (e.g., Finn et al., 2014). In addition to the left inferior frontal regions and the precentral regions, the struggling readers showed reliable activation patterns in the right lingual gyrus and bilaterial visual cortex. Interestingly, no activation was observed in the left fusiform gyrus. Instead, significant activation was observed in the superior parietal cortex, which was not observed in the typical readers. The inclusion criteria for the struggling readers in these studies were based on family history, clinical diagnosis, and/or low performance on standardized reading measures. The distributed activation pattern in struggling readers may reflect the heterogeneous behavioral characteristics in the sample. However, these findings also converge with previous research pointing to hypoactivation in the left occipito-temporal region in struggling readers, a region that supports automatic word recognition. These findings support the notion that individuals with reading (and spelling) disorders may show weaker functional and structural connectivity, thereby reflecting degraded access to phonetic representations (Boets et al., 2013).

Across all the studies, the participants were matched on demographics and background aside from their reading performance. Therefore, in the present study, we examined activation patterns that were common across the two groups to investigate their convergence and divergence in activation patterns. Two regions that both typical and struggling readers recruited to support reading-related activity were the left inferior frontal regions and the left precentral area. One implication of the significant activation observed in the left inferior frontal region may suggest top-down cognitive control relevant to reading. Recent findings offer empirical support for the importance of executive functions and other neurocognitive functions for reading (Cutting et al., 2009; Menghini et al., 2010). There is also evidence suggesting functional heterogeneity within the LIFG. For instance, Wright et al. (2011) reported that activation in BA 44 was associated with lexical decision demands while activation in BA 47 was related to tasks requiring an overt motor response. In the present analysis, both typical and struggling readers showed converging activation in BA 44 (refer to **Table 3**), indicating functional convergence of lexical retrieval demands in the tasks across studies.

Another overlapping region between the two groups was in the precentral area. Aside from the possibility that this region is recruited to prepare behavioral responses during tasks, it is possible that activation in the precentral area is relevant to responsiveness of auditory information (Monzalvo et al., 2012). In Monzalvo et al.’s (2012) study, children with dyslexia showed lower responses to speech in the supplementary motor area, left insula, and posterior temporal cortex. Therefore, it is possible that these are critical regions involved in the auditory processing of speech sounds or that individuals with dyslexia responded less frequently compared to their peers. A recent meta-analysis with adults also demonstrated the importance of the insula as a functional area supporting language production, comprehension, and repetition (Ardila et al., 2014). In the present analysis, struggling readers showed activation in the insula and supplementary motor area, but not in the posterior temporal cortex. In the context of previous work, this finding suggests that struggling readers may not consistently recruit these regions to support phonological processes and reading.

The observation of common regions of activation across the two groups suggested that typical and struggling readers showed convergent activation patterns in anterior regions when engaging in reading-related tasks. However, it was possible that typical and struggling readers have differential magnitudes of activation even though the same brain regions were recruited in reading-related activity (Richlan et al., 2009, 2011). In addition, the divergent patterns of activation in the posterior regions suggested that struggling readers may experience difficulty in processing print as sensory information, particularly in the process of transforming graphemes (as visual stimulation) to phonemes (as auditory information). Successful reading in alphabetic languages relies on a network of brain regions. This was observed in both typical and struggling readers. Building on existing neuroimaging research on neural correlates of reading-related skills, seed-based functional connectivity analysis has great potential to further current knowledge on neural networks supporting successful reading (e.g., Finn et al., 2014). Results from the present analyses could be utilized as data-driven seeds supplementing the identification of seeds based on an *a priori* theoretical approach. Such efforts can indicate not only which distinct regions are relevant for reading, but also how regions operate as a network of coactivated regions in time. Understanding the network properties of the typical and atypical reading brain has the potential to inform practice in several ways: mechanisms underlying reading difficulty can be further elucidated; developmental responses in establishing integrity of reading networks can be determined; and different pathways for remediation and compensation can be examined. Targeted questions for further study should investigate the efficacy of different reading programs according to factors related to the program (i.e., focus of remediation) and to the students (e.g., reading disability characteristics, age, socioeconomic status, and cognitive abilities).

### LIMITATIONS AND FUTURE RESEARCH DIRECTIONS

The present analyses were limited in a few ways that can inform future research directions. First, while we included only studies involving reading in alphabetic languages, there may be language-specific considerations that limit our findings. Across different alphabetic languages, heterogeneous entropy measures have been observed between grapheme–phoneme mappings (Borgwaldt et al., 2004, 2005). We also acknowledge that phonological processing may be modulated by orthographic transparency of alphabetic languages as observed in typical readers (Ziegler et al., 2010) and struggling readers (Landerl et al., 2013). In fact, a recent fMRI study has demonstrated that English readers showed differential activation patterns in superior temporal gyrus when compared to readers of a more transparent language, Dutch (Holloway et al., 2013). Therefore, future meta-analysis on typical and struggling readers involving non-alphabetic scripts may complement findings in the present study.

Second, the present findings are limited by the variance in age groups and diverse experimental and baseline tasks across the included studies. The ages of typical and struggling readers were matched within individual studies, but participant age spanned a wide developmental range within each group of readers. As a result, the meta-analytic results for each group may not correspond to developmental or experiential patterns. As discussed above, qualitative differences in the activation of posterior brain regions were observed across groups. However, we acknowledge that the specific posterior brain regions that are recruited or the degree of activation of these regions is dependent on age, reading experience and tasks (Pugh et al., 2000; Shaywitz et al., 2002; McCandliss and Noble, 2003; Blau et al., 2010; Maurer et al., 2011; Richlan et al., 2011).

The current meta-analyses investigated regions of activation for typical and struggling readers when they were engaged in reading-related tasks, across alphabetic languages. Building on prior work, we conducted separate meta-analyses for typical and struggling readers and through qualitative comparison identified both divergent and convergent patterns of activation. In line with prior research, typical readers showed left lateralized activation in frontal and temporal areas, while struggling readers showed diffuse activation in bilateral frontal, temporal, and occipital regions. In addition, we found convergent regions of activation in the left inferior frontal and precentral areas. With a highly complex behavior such as reading, the learning experience refines the orchestration of a network of brain regions. Based on the results in the present study, reading difficulty is associated with a disruption in the functional activation patterns of key components in the reading brain network. However, the mechanism underlying this functional disruption and how it relates to behavior requires further investigation, considering a developmental framework. Future research taking a network approach investigating the relationship between brain function, brain structure and behavior will shed light on how typical and atypical reading develops. More importantly, designing innovative interventions or support systems for struggling readers may benefit from understanding the similarity and differences in brain and behavior.

## Conflict of Interest Statement

The authors declare that the research was conducted in the absence of any commercial or financial relationships that could be construed as a potential conflict of interest.
